# Effect of Bone Health Optimization on Osteoporosis Screening and Treatment Before Thoracolumbar Fusion

**DOI:** 10.5435/JAAOSGlobal-D-21-00253

**Published:** 2022-03-15

**Authors:** James T. Bernatz, Alec E. Winzenried, Kristyn J. Hare, Anthony L. Mikula, Seth K. Williams, Neil C. Binkley, Paul A. Anderson

**Affiliations:** From the Department of Orthopedics & Rehabilitation, University of Wisconsin School of Medicine and Public Health, Madison, WI (Dr. Bernatz, Dr. Winzenried, Ms. Hare, Dr. Williams, and Dr. Anderson); the Department of Neurological Surgery, Mayo Clinic, Rochester, MN (Dr. Mikula); and the Divisions of Endocrinology and Geriatrics, Department of Medicine, University of Wisconsin School of Medicine and Public Health, Madison, WI (Dr. Binkley).

## Abstract

**Objective:**

Osteoporosis is not rare in thoracolumbar spine fusion patients and may portend poorer surgical outcomes. Implementation of a bone health optimization (BHO) clinic improves osteoporosis screening and treatment in the total joint arthroplasty population. We hypothesize that preoperative osteoporosis is common, under-recognized, and undertreated in thoracolumbar fusion patients and that a BHO clinic will increase preoperative osteoporosis screening rates and pharmacologic osteoporosis treatment in this population.

**Methods:**

This retrospective case series includes adults older than 30 years who underwent elective thoracolumbar spine fusion at a single tertiary care center before and after creation of a BHO referral clinic. Data collected included preoperative osteoporosis risk factors, prior dual-energy radiograph absorptiometry testing, and prior osteoporosis pharmacotherapy. Fracture risk was estimated using the fracture risk assessment tool with and without bone mineral density (BMD), and the US National Osteoporosis Foundation criteria for screening and treatment were applied.

**Results:**

Ninety patients were included in the pre-BHO group; 53 patients met criteria for BMD measurement, but only 10 were tested within 2 years preoperatively. Sixteen patients (18%) met criteria for osteoporosis pharmacotherapy, but only 5 of the 16 (31%) received osteoporosis medication within 6 months of surgery. There were 87 patients in the post-BHO group, and 19 were referred to the BHO clinic. BMD measurement was done in 17 of the patients (89%) referred to the BHO clinic compared with 10% for those not referred. All patients (n = 7) referred to the BHO clinic meeting treatment criteria received treatment within 6 months before surgery, whereas only 25% of the patients not referred received treatment.

**Discussion:**

Osteoporosis is not rare in adults undergoing thoracolumbar spine fusion with ∼13% to 18% meeting criteria for pharmacotherapy. Preoperative BHO referral increases screening and treatment.

Spine fusion for lumbar degenerative disk disease in the United States increased 2.4-fold from 2000 to 2009 and continues to grow.^1,2^ Technologic advances in the past 20 years have led to the development of multiple new fusion constructs (e.g., anterior interbody cages, minimally invasive transforaminal interbody cages, and posterior percutaneous pedicle screws). However, structural integrity of the host bone remains an important factor in construct stability, fusion rates, and failure rates.^3,4^ Accordingly, preoperative identification and perioperative management of osteoporosis are of paramount importance.

Multiple database studies have reported osteoporosis prevalence in those undergoing spinal surgery. Using a Nationwide Inpatient Sample, Guzman et al^5^ found osteoporosis in those undergoing cervical spine surgery to be only 2%. In a study on lumbar spine fusion in the State Inpatient Database, Jain et al^6^ reported osteoporosis prevalence to be 6%. However, a common shortcoming of database studies is that osteoporosis is often under-reported by the International Statistical Classification of Diseases coding. The burden of osteoporosis in spine surgery is, therefore, likely underestimated. Consistent with this, in single-center studies, osteoporosis has been reported as high as 10% in posterior lumbar fusion and scoliosis populations.^3,7^

Previous studies have used World Health Organization criteria based on dual-energy radiograph absorptiometry (DXA) scanning, with osteoporosis defined as a bone mineral density (BMD) T-score of less than or equal to −2.5 and that for osteopenia between −1 and −2.5. However, others recommend osteoporosis be diagnosed based on fragility fractures or fracture risk estimates using the fracture risk assessment tool (FRAX ).^8,9^ Using this approach, osteoporosis is substantially more common in elective orthopaedic surgical patients than that noted earlier being present in approximately 25% before hip or knee arthroplasty^10^ and 32% before shoulder arthroplasty (unpublished data). Studies of osteoporosis prevalence using this broader definition have not been conducted in preoperative spinal surgery patients.

Health optimization interventions are common before elective surgery. Indeed, spine surgeons are optimizing preoperative anemia, body mass index, nutrition, pain control, smoking, and other modifiable factors to decrease postoperative complications.^11^ Because osteoporosis is associated with increased risk of intraoperative and postoperative complications, it is logical that bone health be assessed and optimized before elective orthopaedic surgery.^12^ As such, bone health optimization (BHO) clinics are increasingly being used as a preoperative consultation service to optimize bone health before various orthopaedic surgeries.^12,13^ Our center made this resource available in 2017 to increase appropriate osteoporosis screening, diagnosis, and treatment in the spine fusion patient population. The utilization and effectiveness of this service have not been reported. We hypothesize that preoperative osteoporosis is common, under-recognized, and undertreated in thoracolumbar fusion patients and that a BHO clinic will increase preoperative osteoporosis screening rates and pharmacologic osteoporosis treatment in this population. This study's goals are to determine (1) osteoporosis prevalence in adults undergoing thoracolumbar fusion based on WHO and updated guidelines of the National Osteoporosis Foundation (NOF) and the National Bone Health Alliance and (2) whether availability of a BHO clinic increases screening and treatment of osteoporosis before spine surgery.

## Methods

We conducted a retrospective review of consecutive cases of thoracolumbar or lumbar spine fusion at a single tertiary care center over two time periods. The first period was January to December 2016 (group A), which was before the availability of a BHO referral clinic (which was initiated in early 2017). The second period was January to December 2018 (group B) after the establishment of the BHO clinic. Preoperative BHO referral was at the spine surgeon's discretion in group B. Surgeons were instructed to refer patients if he/she had concerns about the bone health of the patient because of age, comorbidities, or medication use (e.g., chronic steroids). Inclusion criteria were any patient older than 50 years who underwent elective spine fusion (single or multiple levels) that involved the lumbar spine alone or the thoracolumbar spine. Adults older than 30 years were also included if they had any of the clinical risk factors listed in FRAX (Table 1). Exclusion criteria included cases of acute trauma (e.g., burst fracture and fracture/dislocation). If a patient had multiple surgeries during the study period, only the first surgery was included in this analysis. The study was granted exemption by the Institutional Review Board.

**Table 1:** Clinical Risk Factors Included in the FRAX Tool^14^

1. Age
2. Sex
3. Height and weight
4. Previous fracture^a^
5. Parent fractured hip
6. Current smoking
7. Glucocorticoid use^b^
8. Rheumatoid arthritis
9. Secondary osteoporosis^c^
10. Alcohol 3 or more units/d
11. Femoral neck BMD, when available (g/cm^2^)

BMD = bone mineral density

a A previous fracture in adult life occurring spontaneously or a fracture arising from trauma, which, in a healthy individual, would not have resulted in a fracture.

b Equivalent to 5 mg prednisolone daily currently or for >3 months in the past.

c Secondary cause of osteoporosis: type 1 diabetes, osteogenesis imperfecta, untreated long-standing hyperthyroidism, hypogonadism or premature menopause, chronic malnutrition, or malabsorption and chronic liver disease.

Electronic medical records (EMRs) were retrospectively reviewed by the lead author for demographics, preoperative osteoporosis risk factors, prior DXA testing, and osteoporosis pharmacotherapy (a prescription within 6 months before or after surgery). BMD (g/cm^2^) and T-score results were extracted by the lead author after independently reviewing DXA for accuracy. Inaccurate DXA results (i.e., improper default identification of bone edges and regions of interest initially missed by the interpreting radiologist) were not included in this analysis.^14^ The lowest T-score among the average lumbar spine, total proximal femur, and femoral neck was recorded. The FRAX score (10-year major osteoporosis-related and hip fracture risks) was calculated with and without BMD when patients had prior DXA scanning. The NOF, National Bone Health Alliance, and United States Preventive Services Task Force (USPSTF) criteria for BMD testing (Table 2) and pharmacologic osteoporosis treatment (Table 3) were applied to all patients. Each patient was assessed and categorized as having met criteria for DXA testing or not. Similarly, each patient was categorized as having met criteria for pharmacologic treatment or not. The term “appropriately screened” was used to describe patients for whom BMD testing was indicated and who had undergone DXA in 2 years before surgery. The term “appropriately treated” was used to describe patients for whom treatment was indicated (Table 3) and who received a prescription for antiosteoporosis pharmacotherapy within 6 months before surgery. Osteoporosis treatment consisted of any prescription for disphosphonates, denosumab, raloxifene, teriparatide, or abaloparatide.

**Table 2:** NOF and USPSTF Guidelines for BMD Screening^8^

Women	Men
All aged ≥ 65 yr	All aged ≥ 70 yr
Younger postmenopausal women and women in the menopausal transition with clinical risk factors for fracture^a^	Age 50-69 yr with clinical risk factors for fracture^a^
History of fragility fracture after the age of 50 yr	
FRAX MOF risk without knowledge of BMD is ≥8.4%	

a Clinical risk factors found in Table 1.

BMD = bone mineral density, FRAX = fracture risk assessment tool, MOF = major osteoporotic fracture, NOF = National Osteoporosis Foundation, USPSTF = United States Preventive Services Task Force

**Table 3:** WHO, NOF, and NBHA NOF Guidelines for Pharmacologic Treatment of Osteoporosis^8^

T-score ≤2.5 at the femoral neck or spine^a^
History of hip or vertebral fracture
T-score between −1 and −2.5 at the femoral neck or spine and a 10-year risk of hip fracture ≥3% or major osteoporotic fracture ≥ 20%

NBHA = National Bone Health Alliance, NOF = National Osteoporosis Foundation, WHO = World Health Organization

a After appropriate evaluation to exclude secondary causes.

Statistical analysis was completed using Microsoft Excel (version 2016). Chi square tests were used to compare categorical values, while continuous variables were evaluated with two-sample Student *t* tests assuming unequal variance. Categorical variables were evaluated with the Fisher exact test if the number of observations was five or less. *P* values less than 0.05 were considered statistically significant.

The authors report no relevant conflicts of interest, and there was no outside funding involved in the project.

## Results

### Demographics and Procedures Conducted

Ninety and 87 patients were included in group A and group B, respectively (Table 4). The average age in both cohorts was 60 years, average BMI was 29, and 60% were females (59% in group A and 61% in group B). Six patients in group A and five in group B had a previous low-energy fracture. The most common procedure done in group A was a combined fusion, and the most common level was low lumbar. In group B, anterior lumbar interbody fusion was the most common procedure done and the most common level was low lumbar.

**Table 4:** Demographics and Procedures Performed Before (Group A) and After (Group B) Development of the BHO Referral Clinic

	Group A (n = 90)	Group B (n = 87)
Females	53 (59%)	53 (61%)
Age (avg, range)	60, 35-78	60, 32-83
BMI (avg, SD)	29.8 (3.1)	29.2 (3.4)
Fusion procedure		
Anterior interbody	22	39
Lateral interbody	2	1
Transforaminal interbody	17	7
Posterior interbody	11	2
Posterolateral instrumented	13	24
Combined^a^	25	14
Level		
Thoracolumbar	4	3
High lumbar (L1-2 and L2-L3)	8	3
Low lumbar (L3-4 and L4-5)	47	40
Lumbosacral (L5-S1)	20	24
Multiple^b^	12	17

BHO = bone health optimization, BMI = body mass index

a Combined refers to more than one fusion technique (e.g., anterior interbody fusion with posterolateral instrumented fusion).

b Multiple refers to fusion spanning multiple regions (e.g., T12-L4).

### Osteoporosis Screening

In group A, 53 of the 90 patients (59%) met one or more criteria for osteoporosis screening (Table 5). Ten of these 53 patients (19%) had been screened with DXA within 2 years before surgery. Three had T-score ≤ −2.5 (osteoporosis), while four had T-score between −1 and −2.5 (osteopenia). In group B, there was a significant increase in osteoporosis screening rate to 46% (*P* < 0.001). Of the 87 patients in group B, 39 met screening criteria and 18 (46%) had DXA within 2 years before surgery. Among the 19 patients from group B referred to the BHO clinic, 18 of the 19 patients (95%) met one or more criteria for osteoporosis screening. Sixteen of these 18 patients (89%) received DXA within 2 years before surgery, and in all cases, this was obtained at the time of the BHO evaluation. Among the group B patients not referred for BHO, 21 of 68 (30%) met one or more criteria for osteoporosis screening. Two of these 21 patients (10%) had been screened with DXA within 2 years before surgery. Combining the two groups, 92 of the 177 patients (52%) met osteoporosis screening criteria.

**Table 5:** Number of Patients Meeting Criteria for Bone Health Screening

Criteria for Screening	Group A (n = 90)	Group B (n = 87)	*P* Value	Total (n = 177)
Age (women > 65 yr and men > 70 yr)	11	8	0.33	19
History of fragility fracture after the age of 50 yr	3	3	1	6
Age > 50 yr with clinical risk factors for fracture^a^	11	7	0.20	18
FRAX MOF (without BMD) ≥ 8.4%	4	4	1	8
Multiple criteria met	24	17	0.14	41
Total	53	39	0.009	92 (52%)
Screened within 2 yr before surgery	10 (19%)	18 (46%)	<0.001	28 (30%)

BMD = bone mineral density, FRAX = fracture risk assessment tool

a Clinical risk factors listed in Table 1.

The median calculated 10-year FRAX fracture risk in group A was 1.3 (SD ±1.1)% for hip fracture and 7.6 (±5.8)% for major osteoporotic fracture (MOF). Thirty patients had MOF greater than 8.4%, but only eight had DXA within 2 years before surgery. In group B, the median calculated hip fracture risk was 1.6 (±1.2)%, while the MOF risk was 8.4 (±7.9)%. Twenty-eight patients had MOF greater than 8.4%, and 16 had DXA within 2 years before surgery.

### Osteoporosis Treatment

**Figure 1:**
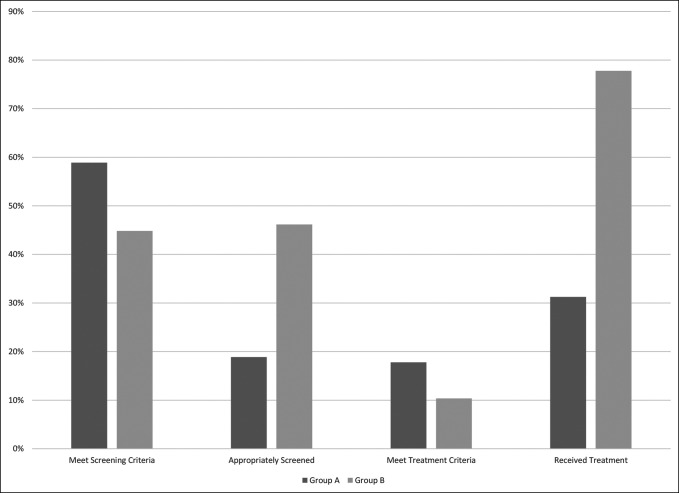
Graph showing the percentage of patients being screened and treated for osteoporosis in group B is significantly (*P* < 0.001) greater than that in group A.

Sixteen of the 90 patients (18%) in group A met NOF criteria for antiosteoporosis pharmacotherapy (Table 6 and Figure 1), with 5 (31%) of them receiving therapy. Three received a diphosphonate, one received denosumab, and one received teriparatide. In the group B patients not referred to the BHO clinic, four of 68 met criteria for treatment, with one (25%) of them receiving treatment (diphosphonate). Seven of the 19 patients (37%) referred to the BHO clinic met criteria for treatment, and all of them were prescribed treatment within 6 months before surgery. Three were prescribed a diphosphonate, three were prescribed teriparatide, and one was prescribed denosumab. Therefore, the appropriate treatment rate for those not referred to BHO clinics was 25%, while those referred to BHO was 100%. The overall increase in treatment between group A and B was statistically significant (*P*< 0.001). Combining groups A and B, the proportion of thoracolumbar fusion patients meeting osteoporosis treatment criteria was 15% (27/177).

**Table 6:** Number of Patients Receiving Indicated Treatment for Antiosteoporosis Medication

Criteria for Treatment	Group A (n = 90)	Group B (n = 87)	*P* Value	Total (n = 177)
Previous low-energy fracture after age 50 yr	5	2	0.17	7
BMD T-score < −2.5	2	1	0.47	3
T-score −1 to −2.5 with FRAX hip > 3% or MOF > 20%	6	2	0.09	8
Multiple criteria met	3	4	0.56	7
Total	16	11	0.26	27 (15%)
Received treatment	5 (31%)	8 (73%)	<0.001	13 (48%)

BMD = bone mineral density, FRAX = fracture risk assessment tool, MOF = major osteoporotic fracture

## Discussion

Poor bone health is common in patients who undergo elective spine procedures and has been linked to poor outcomes and complications.^13^ These complications include implant failure, fracture, kyphosis, subsidence of interbody devices, proximal junctional fracture, and fusion failure.^15-17^ Bjerke et al.^3^ found that osteoporosis-related complications correlated with severity of bone disease: 50% of the patients with osteoporosis had complications, 34% of the patients with osteopenia had complications, while only 23% of the patients with a normal bone quality had complications. This study finds the prevalence of osteoporosis in adults undergoing thoracolumbar fusion to be 15%. This is likely an underestimation because not all patients were screened with DXA. In the group of patients who were referred for BHO, the prevalence of osteoporosis was 37%. This may represent referral bias but suggests that the 15% reported prevalence here is lower than the true prevalence.

The availability of a fracture liaison service in our system in late 2016 opened the possibility of preoperative BHO. The principles of secondary fracture prevention and BHO are similar. The availability, improved access, and knowledge regarding bone health resulted in a notable increase in overall screening and treatment of preoperative spine surgery patients. Furthermore, a referral for BHO resulted in comprehensive screening and recommendations of treatment universally when warranted. Thus, similar to the results of fracture liaison service programs, our BHO program led to notable improvements in the assessment and management of bone health. However, the success of the BHO program relies on the surgeon referral. In group B patients not referred to BHO, the appropriate treatment rate was only 25% vs 100% for those referred to BHO. Therefore, the surgeon plays an important role in initiating the BHO process.

The International Society of Clinical Densitometry recently released official position statements on the use of bone health evaluation in orthopaedic surgery.^18^ Briefly, these recommendations include consideration of bone health assessment in patients before elective spine surgery. Moreover, men older than 70 years, women older than 65 years, and any patient with the following conditions are at greater risk for impaired bone health and should have DXA testing: diabetes mellitus, inflammatory arthritis, chronic corticosteroid exposure, low-trauma fracture after age 50 years, chronic kidney disease, limited mobility, and smoking. Identification of high-risk patients should be undertaken by spine surgeons and/or their clinical staff and can prompt referral to a bone health specialist. We recommend the NOF and USPSTF guidelines as a reference for identifying patients who warrant DXA screening and/or BHO referral. In this study, referral to the BHO clinic increased DXA screening rates from 19% to 89% and presurgical osteoporosis treatment rates from 31% to 100%, which were both statistically significant. This study was not designed to detect differences in clinical outcomes between these groups but rather to identify the incidence of osteoporosis and to highlight the improvement of screening and treatment with a BHO referral clinic.

FRAX, a fracture risk estimation tool, was developed in cooperation with the WHO and estimates 10-year risk of MOF and hip fracture based on well-validated fracture risk factors.^19^ In addition to the clinical risk factors discussed earlier, FRAX can be used to aid in identifying those patients who should receive osteoporosis screening or treatment. Importantly, FRAX without BMD does not require any additional diagnostic testing, making it a practical means of identifying patients who should undergo DXA testing. In orthopaedic patients, Kadri et al.^12^ found that the 10-year MOF risk was not markedly different with or without BMD, thus demonstrating the potential usefulness of this screening tool. In this regard, the USPSTF recommends obtaining DXA when the 10-year MOF risk by FRAX without BMD is ≥8.4%. This threshold was set because it is the risk of a 65-year-old woman in the United States without additional risk factors for fracture. Given the absence of evidence, we have selected this value; however, it is unknown whether this provides appropriate sensitivity and specificity to identify presurgical patients who should have DXA done.

Osteoporosis poses the risk of bone-related complications in spine surgery. Early postoperative complications (<3 months from surgery) including pedicle fractures and compression fractures have been reported in 13% of the patients with a poor bone quality.^20^ Liu et al.^21^ found an increased risk of skeletal complications and revision surgery in those with low volumetric BMD. Late (>3 months from surgery) complications can include subsidence of interbody cages, proximal junctional failure, screw loosening, adjacent segment degeneration, pelvic insufficiency fractures, and instrumentation failure.^22-24^ The first step in mitigating these complications is identifying those at higher risk. We report the notable improvement in screening and preoperative treatment of osteoporosis after initiation of a BHO referral clinic.

Multiple previous studies have identified the potential benefits of disphosphonates and anabolic agents in spine fusion. The antiresorptive disphosphonates are a more widely prescribed first-line treatment for osteoporosis, whereas teriparatide/abaloparatide is an alternative medication that increases BMD by activating osteoblasts more than osteoclasts.^25^ In comparative studies, teriparatide has been found to increase fusion rates compared with control,^26^ whereas disphosphonates have not.^27-29^ Teriparatide has been associated with decreased risk of screw loosening as well (27% in control vs 10% in teriparatide group).^30^ Diphosphonate use was associated with decreased odds of vertebral fractures at the fusion segments or adjacent segments compared with control subjects.^27,29,31^ Functional outcomes between fusion patients receiving teriparatide or diphosphonate and controls have been equivocal.^29,30,31,32^ Additional studies are needed to examine long-term functional outcomes, risk of proximal junctional kyphosis, adjacent segment degeneration, and need for revision surgery.

This study has multiple limitations. First, the study was not designed or powered to identify differences in clinical outcomes between groups A and B. In addition, the EMR can often under-report comorbid conditions that may increase fracture risk. Therefore, the reported prevalence of osteoporosis in preoperative spine fusion patients is likely underestimated. Osteoporosis screening and treatment undertaken elsewhere (i.e., another state) were also not captured in this study. A family history of hip fracture, which is a component of the FRAX calculation that can strongly affect the calculated risk, is not well recorded in the EMR. Our cohort was predominantly White and represents the population served by a tertiary referral center, limiting the potential generalizability to community-based practice or those of other ethnic populations. It should also be highlighted that this study was not designed to address the questions of when to initiate pharmacotherapy preoperatively, how long to continue pharmacotherapy postoperatively, or whether surgery should be delayed. We think these to be important areas of future study.

Our study finds that osteoporosis is common, underevaluated, and undertreated before thoracolumbar fusion. The initiation of a BHO referral clinic can increase the rate of screening and treatment before surgery. The use of available screening guidelines (NOF and USPSTF) can help surgeons determine which patients may need referral for screening and treatment, if indicated. Therefore, spine surgeons should be aware of osteoporosis risk factors and, when the resource is available, refer these patients to bone health specialists.
